# Proof of concept: real-time viability and metabolic profiling of probiotics with isothermal microcalorimetry

**DOI:** 10.3389/fmicb.2024.1391688

**Published:** 2024-06-19

**Authors:** Carlotta Morazzoni, Madle Sirel, Serena Allesina, Marta Veses Garcia, Kasper Kragh, Marco Pane, Katrin Beilharz

**Affiliations:** ^1^Probiotical Research s.r.l., Novara, Italy; ^2^Symcel AB, Stockholm, Sweden

**Keywords:** viability assessment, viability enumeration, metabolic activity, beneficial microbes, probiotics, real-time, isothermal microcalorimetry

## Abstract

Isothermal microcalorimetry (IMC) is a potent analytical method for the real-time assessment of microbial metabolic activity, which serves as an indicator of microbial viability. This approach is highly relevant to the fields of probiotics and Live Biotherapeutic Products (LBPs), offering insights into microbial viability and growth kinetics. One important characteristic of IMC is its ability to measure microbial metabolic activity separately from cellular enumeration. This is particularly useful in situations where continuous tracking of bacterial activity is challenging. The focus on metabolic activity significantly benefits both probiotic research and industrial microbiology applications. IMC’s versatility in handling different media matrices allows for the implementation of viability assessments under conditions that mirror those found in various industrial environments or biological models. In our study, we provide a proof of concept for the application of IMC in determining viability and growth dynamics and their correlation with bacterial count in probiotic organisms. Our findings reinforce the potential of IMC as a key method for process enhancement and accurate strain characterization within the probiotic sector. This supports the broader objective of refining the systematic approach and methods used during the development process, thereby providing detailed insights into probiotics and LBPs.

## 1 Introduction

In recent years, the precise assessment and quantification of microbial viability has become increasingly important, especially in the microbial biotechnology sector. This sector encompasses various activities such as research and development, product formulation, and quality control of probiotics and Live Biotherapeutic Products (LBPs). Although considered the gold standard for viability assessment, traditional methods such as plate count enumeration present significant drawbacks. These methods are laborious and time-consuming, requiring incubation times that can extend from 2 to 5 days, depending on the microbial strain and the sample matrix (Fredua-Agyeman and Gaisford, 2015). Moreover, plate counts are susceptible to intrinsic variability, often within a range of 20–30%, which can significantly impact the accuracy of results (Jongenburger et al., 2010; Hansen et al., 2018). An additional concern is that certain microorganisms, despite being metabolically active, may fail to form colonies on standard agar plates, thus potentially leading to an underestimation of viable cell count (Staley and Konopka, 1985; Kell et al., 1998). Given these limitations, it has become evident that bacterial cultures exhibit heterogeneity, where viability is not strictly a function of the ability to replicate. The conventional plate count method may not effectively capture the entire spectrum of microbial activity, particularly for those cells termed “viable but non-culturable” (VBNC). This has driven the exploration of alternative methods that can provide a more comprehensive and nuanced picture of the microbial viability (Boyte et al., 2023).

For real-time monitoring of bacterial activity, pH monitoring has traditionally played an important role, especially as a complementary method for viability assessment. Acidification rates serve as a metric for real-time bacterial growth monitoring, particularly applicable for organic acid-producing organisms like lactic acid bacteria (LABs) (Visciglia et al., 2022).

Most commercially available probiotic strains trace their origins to the food and dairy industry and fall within the fermenting organic acid producers. The probiotics market is undergoing rapid growth, introducing new strains with beneficial probiotic properties that are used as Next Generation Probiotics (NGPs) or LBPs (O’Toole et al., 2017; Martín and Langella, 2019). Many of these strains are difficult to evaluate using conventional methods because they require strict anaerobic conditions or have proteolytic properties that interfere with methods like pH monitoring, optical density (OD) measurements, and plate counting (Wendel, 2021; Boyte et al., 2023). Two-step approaches, combining a less precise solution with a high-resolution method, offer a balance between economic constraints and higher throughput (Berninger et al., 2018). For instance, culture-based methods such as isothermal microcalorimetry (IMC) can serve as an initial screening step, enabling the evaluation of various formulations with minimal restrictions and higher throughput. This approach goes beyond a time or endpoint assay by providing kinetic information in addition to viability quantification, aiding in the selection of optimal culture conditions and formulations.

**FIGURE 1:**
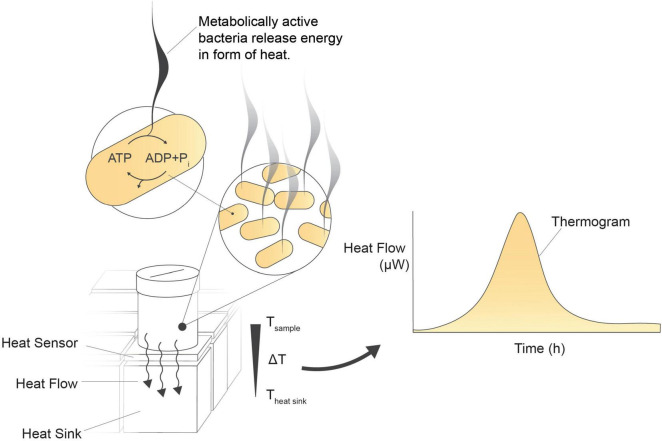
Isothermal microcalorimetry (biocalorimetry) principle. Isothermal incubation of a microbial culture in a vial positioned above a heat sensor on a heat sink. As metabolically active cells release energy, a byproduct of metabolic pathway reactions, the generated heat disperses over the heat sensor toward the heat sink. The heat sensor detects and converts these minute heat changes into an analog signal in μW. Heat flow is continuously measured every 2 s, enabling real-time monitoring of the sample’s activity.

This study explores the complexities of microbial viability assessment, spotlighting the often overlooked technique of IMC (Braissant et al., 2015b; Garcia et al., 2017; Berninger et al., 2018; Nykyri et al., 2019). IMC monitors heat generation by a sample over time while maintaining a constant temperature (Figure 1). This heat originates primarily from the metabolic processes of microorganisms, which are essentially biochemical exothermic reactions. IMC is a highly sensitive method that captures even the slightest changes in heat produced by living organisms. The resulting heat flow curve (μW/s) directly reflects the metabolic rate of the microorganisms, making it an effective approach for viability assessment (Mihhalevski et al., 2011; Braissant et al., 2015a; Garcia et al., 2017; Fredua-Agyeman and Gaisford, 2019; Nykyri et al., 2019).

Relying on the total heat flow generated by the entire population in a sample, IMC is applicable to diverse sample types and media matrices. Notably, it remains unaffected by turbidity and viscosity and is insensitive to cell clumping (Braissant et al., 2010, 2015b). Despite the method’s versatility and the valuable insights IMC can offer into microbial viability, the lack of case studies and the scarcity of comparative data have been hindering factors in implementing IMC in the probiotics industry.

This article introduces a novel method for viability assessment that decouples microbial cellular quantification from metabolic activity assessment using IMC. Emphasizing its wide applicability beyond cellular enumeration, this method includes assessing metabolic activity and growth under specific conditions by comparing it to complementary data from other methods, such as flow cytometry. IMC’s versatility positions it as a valuable tool for developing new live bacteria products, growth media compositions, formulations, and stress tolerance.

## 2 Materials and methods

### 2.1 Microorganisms and culture conditions

*Lactobacillus plantarum* 299v (Probi AB) was utilized to evaluate method suitability, with inoculation commercial product powder formulation containing lyophilized cells and cryostocks of the isolated strain. Two inoculation methods were employed. First, the full capsule content of a commercial product (approximately 370 mg, claimed to contain at least 10 billion bacteria) was resuspended in 10 ml of peptone water (casein peptone 10 g/L, sodium chloride 5 g/L) and revived at room temperature for 30 min. Second, fresh colonies from de Man-Rogosa-Sharpe (MRS) agar plates were used to inoculate 2 ml of MRS (Millipore) in 15 ml plastic conical tubes. These tubes were capped and incubated overnight at 37°C and rotated at 180 r.p.m.

For the metabolic activity study using acidification kinetics, cytofluorimetric enumeration, and IMC in parallel, two strains, *Lacticaseibacillus rhamnosus* GG ATCC 53103 and *Limosilactobacillus fermentum* LF10 DSM 19187, both from the Probiotical SpA collection, were used. The probiotic strains were activated overnight at 37°C in MRS (Difco, BD, MD) broth and then sub-cultured, using at least two passages over the mid-log phase.

### 2.2 Microcalorimetric analysis for viability enumeration

A 10-fold dilution series of the bacterial suspensions of *L. plantarum* in peptone water was prepared. The isothermal microcalorimeter plate, equipped with titanium vials and plastic inserts, was prepared by adding 30 μl of the individual sample dilutions to 270 μl of sterile MRS broth. The vials were sealed and placed into a calScreener™ isothermal microcalorimeter (Symcel AB, Sweden) in accordance with the manufacturer’s guidelines. Kinetic heat flow was monitored using the calView 2.0 software (Symcel AB, 2023), which recorded heat flow curves over a period of 48 h. Various parameters were extracted using the online analysis tool calData.^1^

To assess the impact of turbid media viability counts, commercial plant-based milk (pasteurized oat drink, Oatly Barista Edition) was used. For culturing, 1:1 dilutions of MRS/oat drink were inoculated with serially diluted *L. plantarum* overnight culture in MRS, and 300 μl of the dilutions were added to vials for calorimetric measurements, as described above.

### 2.3 Viable counts using plate count assay

de Man-Rogosa-Sharpe agar plates were used to determine counts of viable colony-forming units (CFUs). Samples were serially diluted in peptone water in 10-fold steps. Of the diluted sample, 50 μl were spread on top of the agar of the plates, and subsequently incubated at 37°C for 48 h. Following incubation the colonies were counted and the viability value (CFU/ml) was calculated.

### 2.4 Cytofluorimetric counts

For strains GG and LF10, the BD Cell Viability Kit (BD Biosciences, Milan, Italy) was utilized to quantify viable cells (AFU/ml) and total cells (TFU/ml). Cell staining procedures were conducted in accordance with ISO 19344: IDF 232 (2015). Briefly, 100 μl of a diluted suspension containing approximately 10^5^–10^6^ cells/ml in buffered peptone water was mixed with 885 μl of PBS. Subsequently, 10 μl of propidium iodide (previously diluted in water at 0.2 mmol/L) and 5 μl of thiazole orange (42 μmol/L) were added to the dilution, followed by vortexing. The CytoFLEX cytometer (Beckman Coulter SRL), equipped with 488 nm laser excitation and CytExpert software, was employed for analysis. Thresholds for side scatter (SSC-H) and forward scatter (FSC-H) were established for microbial cells, which were gated using forward versus side scatter (FSC-H vs. SSC-H). The optimal discrimination between live and dead populations, used for the enumeration of AFU and TFU, was achieved on an FL1 versus FL3 plot. No counting beads were added to the diluted samples as internal standards as the instrument is designed for volumetric counting, whereby the concentration of events (cells) is determined based on the defined sample volume drawn by the needle.

### 2.5 Analysis of metabolic activity

The overnight cultures of GG and LF10 were inoculated in 30 ml of three growth media of different formulations (medium A, medium B, and medium C), with a final concentration of 10^7^ AFU/ml.

For medium A, we employed MRS as the standard reference for the growth of Lactobacilli. Media B and C had two distinct formulations, each intentionally designed with differences in salt composition and primarily focusing on variations in carbon source concentration and nitrogenous source composition. The standardized cultures were placed at 37°C in a water bath for 24 h and pH variation during fermentation was measured at intervals of 4 min with the iCinac pH monitoring system (KPM Analytics, USA). At fixed time points (*t* = 0, 3, 5, 7, and 24 h) an aliquot of sample was taken and analyzed by CytofFLEX cytometer, as described above.

Simultaneously, microcalorimetric analysis was performed by adding 10^7^ AFU in each vial provided with a plastic insert and 300 μl of the three different media. As described in section “2.2 Microcalorimetric analysis for viability enumeration,” kinetic heat flow was monitored over a period of 48 h and heat flow curves were generated using the calView 2.0 software. Various parameters were extracted using the online analysis tool calData.

### 2.6 Statistical analysis for standard curve base viability enumeration

Standard curves were generated using both freshly prepared and freeze-dried cells. The initial cell concentration for fresh cells was approximately 3 × 10^9^ CFU/ml. For rehydrated freeze-dried cells the concentration was approximately 10^10^ CFU/ml. The time to peak (TTP) values for each sample (liquid and freeze-dried) were calculated as a viability marker, and GraphPad Prism 10.0.2 (232) was employed for further analysis. The regression curves were constructed by plotting TTP against the logarithm of the cell concentration (CFU/ml). To analyze the reproducibility of IMC for viability assessments an unpaired two-tailed *t*-test was used in the same software.

## 3 Results

### 3.1 Assessing reproducibility of isothermal microcalorimetry for viability assessment

To examine the suitability of IMC for viability assessment and enumeration of probiotic products, we utilized a 48-channel isothermal microcalorimeter, the calScreener™, to test the reproducibility of the method.

**FIGURE 2:**
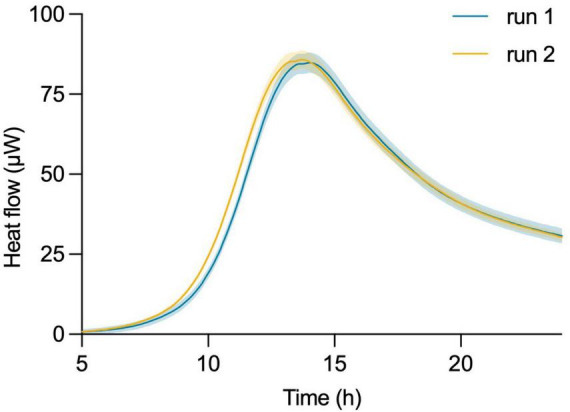
Robustness and reproducibility of isothermal microcalorimetric measurements using the calScreener™. Thermograms of two independent experiments on different days of *L. plantarum* started from independent batches of commercial probiotic product. Shading of each curve shows the SD of the 32 individual replicates per experiment.

In this assessment, a freeze-dried commercial probiotic product containing *L. plantarum* was diluted in fresh MRS medium and evenly distributed into 32 titanium vials. This plate was then introduced into the microcalorimeter, where over 24 h the instrument continuously monitored the heat flow from the samples and generated heat flow curves (thermograms). Notably, the readings from the individual sample vials gave consistent results, as indicated by the minimum SD (range 2.906–0.03 μW for the collected data points) of the thermograms. The same experiment was repeated on a different day, using a different instrument and a new batch of freeze-dried bacteria from the same product (Figure 2). An unpaired two-tailed *t*-test revealed no statistically significant difference [*t*(582) = 0.0258, *p* = 0.9794] between the two groups. This indicates that the observed difference in the means of the two thermograms is likely due to random chance and not a true underlying effect.

### 3.2 Establishing a standard curve for viable cell enumeration

**FIGURE 3:**
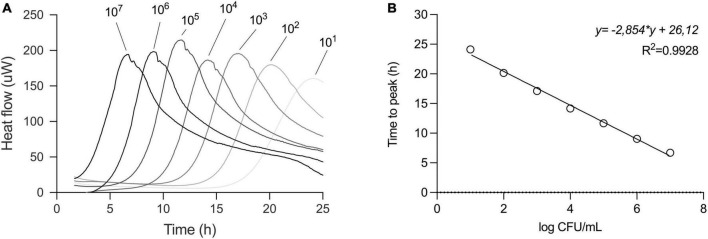
Serial dilution of *L. plantarum* to build a standard curve. **(A)** Thermograms of serial dilution of overnight culture of *L. plantarum* in MRS broth. Initial inoculum sizes are depicted above the thermograms. **(B)** For standard curve, the time to peak (TTP) of each dilution from the thermogram was determined and values were plotted against CFU/ml of respective dilution as confirmed by plate count. Relationship between TTP and CFU was described in a linear regression model.

In our efforts to quantify the viability of *L. plantarum*, we conducted a series of experiments involving serial dilutions from overnight cultures, which were then inoculated into fresh MRS medium for isothermal calorimetric measurements at 37°C. The resulting thermograms consistently showed the thermal fingerprint of *L. plantarum* emerging at predicted intervals, confirming the method’s suitability. There was a linear relationship between the initial inoculum size and the appearance of the thermograms (Figure 3A). We chose to analyze the data using the time to metabolic peak parameter to illustrate the principle. Time to signal detection, another calorimetric parameter extracted from the thermograms, exhibited a linear relationship with an initial number of microbial cells. Determining this parameter involved applying a threshold value set at a certain μW value to the data.

In parallel, the initial cell number was determined by plate count assay and a calibration curve was plotted. To create a standard curve for inoculum estimation, we applied a linear regression model to the data.

This approach allowed us to obtain a standard curve specific to strain under specified conditions, such as medium (here MRS), volume, and temperature (Figure 3B).

**FIGURE 4:**
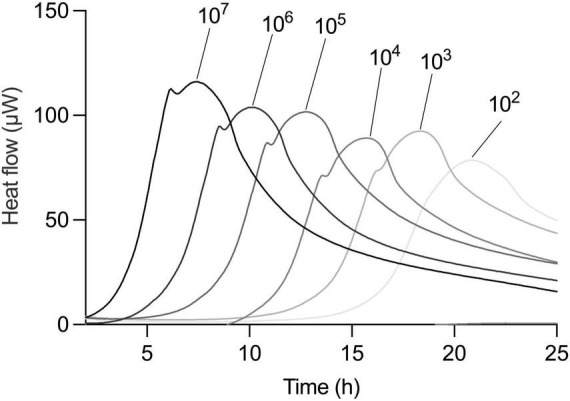
Correlation of TTP and CFU is universal independent from medium type. Metabolic activity of serial dilution of an overnight culture of *L. plantarum* in 1:1 MRS and oat drink. Dilution factors are indicated above thermograms.

To further challenge the method’s robustness, we investigated whether the linear relationship between inoculum size and TTP held true under different pre-culture and incubation conditions. We first used a dilution series of *L. plantarum* in a one-to-one MRS medium-oat drink mixture. The thermogram profiles remained consistent under these defined conditions, exhibiting the expected rightward shift with lower initial bacterial loads (Figure 4). Importantly, the strong linear relationship between inoculum size and TTP was maintained (Table 1).

**TABLE 1:** Summary of linear regression results of the five independent standard curve experiments.

*L. plantarum* suspension	Regression equation (*Y* value)	*R* ^2^
**MRS broth**		
Fresh culture	−2.854x + 26.12	0.9928
Freeze dried 1	−2.736x + 25.92	0.9977
Freeze dried 2	−2.722x + 26.05	0.9988
Freeze dried 3	−2.941x + 27.38	0.9900
**MRS: oat drink**		
Fresh culture	−2.702x + 26.33	0.9997

**FIGURE 5:**
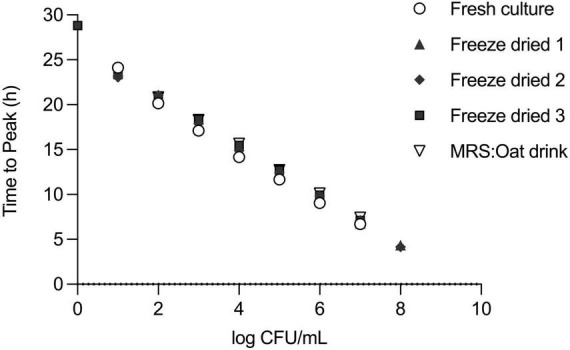
Reproducibility of standard curve under different pre-culture and incubation conditions of *L. plantarum*. Step-wise dilutions of *L. plantarum* (10^9^ CFU/ml) overnight culture in MRS or a one-to-one MRS medium-oat drink mixture or step-wise dilution of reconstituted powder of commercial freeze-dried formulation of *L. plantarum* in MRS. Measurement by IMC and confirmation of initial cell number by plate counting. For the standard curves, TTP and CFU values were plotted on *x*–*y* axes.

Secondly, we explored the impact of pre-culture conditions by using three independently processed, freeze-dried samples. The standard curves generated from these samples closely resembled those obtained with the original liquid culture (Figure 5). As expected, the linear regression models for all three independent experiments again demonstrated a strong linear relationship between inoculum size and TTP (Table 1). To further solidify this finding, an F-test revealed no statistically significant difference between the slopes of the five linear regressions (*F* = 1.569, DFn = 4, DFd = 27, *P* = 0.2111), reinforcing the method’s robustness across culture conditions.

Generalizing this standard curve across a broader range of strains and growth conditions may require further validation.

### 3.3 Beyond viability enumeration: IMC as a complementary approach

Empirical data involving various media formulations has shown that combining biomass quantification using time point measurements and metabolic activity measurements provide a comprehensive view of microbial activity and viability under diverse conditions. Especially during the substrate or media optimization process, it is relevant to consider various parameters.

**FIGURE 6:**
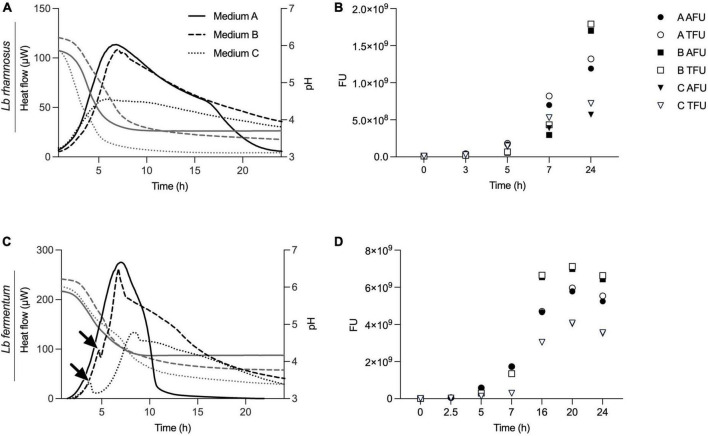
Real-time metabolic activity measurements to complement acidification and cell count data. Three growth medium formulations, medium A, B and C, were evaluated using acidification curves, isothermal microcalorimetry (plotted in panels **A**,**C**) and time point measurements using flow cytometry (shown in panels **B**,**D**). Two different strains, *L. rhamnosus* and *L. fermentum*, were used for the experiments. IMC data are presented as heat flow in μW, acidification curves depict pH values and for flow cytometry fluorescent units (FU) with total FU (TFU) and alive FU (AFU) are displayed.

Here, we executed three different experiments – IMC, flow cytometry for viability enumeration, and acidification measurements – in parallel on two representative probiotic strains, *L. rhamnosus* and *L. fermentum* (Figure 6). We demonstrated how information obtained by isothermal calorimetry, such as metabolic activity (heat flow over time) and peak metabolic activity gives an indication of viability, with information on growth, metabolic dynamics, and biomass formation during medium fermentation.

Unlike IMC, flow cytometry does not permit kinetic measurements and samples are taken at different time points. For the measurements, cells were sampled at *t* = 0, 3, 5, 7, and 24 h and viable counts expressed as active fluorescent units (AFU) (Figures 6B, D). When compared to thermograms, there was a correlation between metabolic activity profiles and biomass data obtained by flow cytometry (Figure 6). In particular, it is evident how the formulation of medium B, properly designed for strain activation, proves to be more active in stimulating and supporting growth compared to the standard medium for *lactobacilli* (MRS) and the third formulation, medium C, which is less effective in reaching high cell counts. For *L. fermentum*, we see a first, smaller metabolic peak at around 3–4 h when cultivated in medium B or C. This indicates a metabolic adaptation phase with a metabolic shift as indicated with arrows in Figure 6C. No such information was revealed in acidification curves, nor from the time point biomass measurements.

Additionally, we see that low biomass formation, early acidification, and higher acidification are associated with lower overall metabolic activity and a reduced metabolic peak. Figures 6C, D illustrate that IMC accurately reflects growth dynamics. For instance, the delayed and lower biomass formation measured by flow cytometry of *L. rhamnosus* in medium C is mirrored in the delayed metabolic activity signal, a nuance not captured by the acidification curves.

## 4 Discussion

Traditional viability assessment in microbial cultures or formulations relies on the gold standard method of plating serial dilutions and determining CFU. However, this method has inherent variability and may overlook aspects of bacterial population heterogeneity. To address these limitations, alternative techniques such as flow cytometry and PCR methods have been introduced, offering the ability to distinguish between viable and dead cells. In this context, IMC stands out as a potent method for viability assessment, offering an innovative and versatile solution that is both sensitive and rapid.

Isothermal microcalorimetry distinguishes itself by measuring the total metabolic activity of the bacterial population even in complex matrices. This has been demonstrated in studies involving *Lactobacilli* and the direct viability assessment of bacterial-coated seeds (Garcia et al., 2017; Nykyri et al., 2019). The real-time monitoring distinguishes IMC from other methods like plate count enumeration and flow cytometry, which provide data at specific time points (Braissant et al., 2010; Fredua-Agyeman and Gaisford, 2019). In our experiments, TTP values were used for standard curve-based enumeration. The time to detect a signal at a chosen detection threshold can also be used, allowing for more rapid detection and making it independent from the thermogram fingerprint (Garcia et al., 2017; Fricke et al., 2019).

In the probiotics industry, strains belonging to *Bifidobacteria* spp. or *Lactobacillus* spp. taxa may pose technical challenges that traditional plate counts cannot easily address. This is particularly true for NGPs or LBPs where the emergence of novel strains presents new challenges, emphasizing the need for improved viability assays. These challenges include difficulties in growth, sensitivity, intolerance to oxygen, and clumping tendencies, which can impact industrial production and viability determination.

Isothermal microcalorimetry’s sensitivity registers minimal changes in heat released from metabolically active cells. Metabolically active bacterial cells produce heat at an approximate rate of 2 pW per cell (Braissant et al., 2010). Operating within the micro-Watt range, IMC can detect as few as approximately 2 × 10^4^–10^5^ actively growing cells at the point of detection (Braissant et al., 2010). The method has also been used to follow microorganisms in different media matrices, such as ground meat, milk, juice, and urine (Gram and Søgaard, 1985; Gunasekera et al., 2000; Alklint et al., 2005; Bonkat et al., 2012; Maskow et al., 2012; Fricke et al., 2019; Nykyri et al., 2019).

Incorporating IMC into process and product development can ease the integration of newly identified beneficial microbes into the development pipeline. This is particularly beneficial for more delicate microbial species that are sensitive to environmental perturbations, obligate anaerobes, or those not engaged in acid fermentation. The minimal sample preparation required, the quick results, and the robustness of the technique against different matrices make IMC an attractive choice (von Ah et al., 2009; Braissant et al., 2010; Garcia et al., 2017). Moreover, IMC can discern the impact of active ingredients on bacterial activity, which may either stimulate or inhibit metabolic processes, a detail that may escape traditional methods like plate counting and modern approaches such as PCR methods and flow cytometry.

The usefulness of IMC is further demonstrated in its ability to quantify the effects of stress conditions encountered by bacteria during manufacturing processes or transit through the gastrointestinal tract, similar to the viability enumeration methods discussed here (Fredua-Agyeman and Gaisford, 2015). A notable application of this was in the study of clinical isolates of *Staphylococcus aureus* subjected to dehydration stress; IMC proved to be a direct and effective tool for quantifying effects on viability. In this study, *S. aureus* isolates were coated on PVDF coupons and then introduced into calorimetric vials for measurement (Baede et al., 2022).

While IMC offers valuable insights into microbial viability, its integration into the probiotics industry is hindered by a relative scarcity of comparative data compared to established techniques such as quantitative PCR, flow cytometry, and plate counting methods. This information gap can pose a deterrent for new users who may find it difficult to validate and trust IMC without a robust dataset. Additionally, the high sensitivity of IMC requires careful consideration of strain variations, media lot differences, and temperature fluctuations, which can significantly impact measurement outcomes. However, these challenges can be effectively mitigated by implementing appropriate controls. Additionally, IMC provides precise metabolic fingerprint for each experiment, readily indicating any deviations from expected patterns. In comparing IMC, flow cytometry, and acidification curves for assessing microbial viability, it can be seen that each method offers distinct advantages. Parallel measurements of flow cytometry and IMC revealed a positive correlation between increasing viable counts and metabolic activity in time. While flow cytometry offers a high resolution in distinguishing live and dead cells at sampled time points, IMC excels in continuous measurement of the overall metabolism of samples.

## 5 Conclusion

In summary, IMC introduces an innovative approach to viability assessment that brings knowledge beyond pure cellular enumeration. Its adaptability and continuous monitoring make it a versatile medium-throughput tool in many steps of the product development journey. Further investigation is needed to fully explore this novel concept.

## Data availability statement

The original contributions presented in this study are included in this article/supplementary material, further inquiries can be directed to the corresponding author.

## Author contributions

CM: Conceptualization, Formal analysis, Investigation, Methodology, Writing – original draft, Writing – review & editing. MS: Formal analysis, Investigation, Methodology, Writing – original draft, Writing – review & editing. SA: Investigation, Methodology, Writing – original draft, Writing – review & editing. MV: Writing – original draft, Writing – review & editing. KK: Writing – original draft, Writing – review & editing. MP: Conceptualization, Writing – original draft, Writing – review & editing. KB: Conceptualization, Project administration, Visualization, Writing – original draft, Writing – review & editing.

## Footnotes

1
https://symcel.com/analysis-tools/calorimetric-growth/
